# 7p21.3 Together With a 12p13.32 Deletion in a Patient With Microcephaly—Does 12p13.32 Locus Possibly Comprises a Candidate Gene Region for Microcephaly?

**DOI:** 10.3389/fnmol.2021.613091

**Published:** 2021-02-04

**Authors:** Martina Rincic, Milan Rados, Janja Kopic, Zeljka Krsnik, Thomas Liehr

**Affiliations:** ^1^Croatian Institute for Brain Research, School of Medicine, University of Zagreb, Zagreb, Croatia; ^2^Jena University Hospital, Friedrich Schiller University, Institute of Human Genetics, Jena, Germany

**Keywords:** microcephaly, magnetic resonace imaging (MRI), intellectual disability—genetics^*^, PRMT8, array comparative genetic hybridization (aCGH)

## Introduction

Genomic regions of copy number changes, gains and losses intermediate in size named copy number variant (CNV) are not only important source of genomic variation in the general population, but are also well-known causes of many neurodevelopmental disorders (Lee and Scherer, 2010; Weise et al., 2012). CNVs can have impact on gene/genes function via several different mechanism (Hehir-Kwa et al., 2013). Generally, assessing the clinical relevance of CNVs in cases with wide phenotypic spectrum can be challenging and should be looked as an ongoing process that can be subject to change over time (Tsuchiya et al., 2009; Hehir-Kwa et al., 2013; Palmer et al., 2014). Concomitant presences of two simultaneous interstitial genomic losses are rare and in most such cases it is difficult to attribute the symptoms to one of the two affected genomic regions. However, two-hit- and compound heterozygosity-models might be important mechanisms underlying the pathogenesis in such cases (Uehara et al., 1993; Gau et al., 2012; Mascelli et al., 2014). Furthermore, selecting a single gene for further functional experiments in case of rear, large or singleton CNV can be doubtable. Recent paper by Yamasaki et al. describes how assessing and combining the influences of gene dosage sensitivity and gene expression sensitivity proper candidate genes could be selected (Yamasaki et al., 2020).

Interstitial deletions involving the distal portion of 12p are rarely reported in literature, while terminal 12p deletions represent one of the rarest subtelomeric imbalances (Ravnan et al., 2006). Cases with terminal or interstitial 12p deletions are presenting a phenotypic spectrum ranging from normal development to prominent growth and developmental delay, facial dysmorphism and microcephaly. Even families with del(12)(p13.31) were attributed as being potentially among the group of unbalanced chromosomal aberrations (UBCA) without clinical impact (Liehr, 2014). Among the most prominent phenotypic feature of 12p deletions is microcephaly (head circumference ≤-3SD). Literature and current databases search for 12p13.32 locus showed that only several cases have been published or reported, so far (Mayeda et al., 1974; Magnelli and Therman, 1975; Romain et al., 1987; Velinov et al., 2008; Madrigal et al., 2012; Vargas et al., 2012; Thevenon et al., 2013; Fanizza et al., 2014; Faria et al., 2016). Early publications (pre-2000s) reporting on del(12)(p13pter) are all based on cytogenetically visible deletions, meaning that exact breakpoints could not be determined; but they all report microcephaly (Mayeda et al., 1974; Magnelli and Therman, 1975; Romain et al., 1987).

Interstitial deletions of 7p21.3 are even rarer and therefore the corresponding phenotypic spectrum is still unknown. Only four cases are reported with overlapping deletion in 7p21.3, by now (Shetty et al., 2007; Liao et al., 2012; Massalska et al., 2014).

Here we report a female patient with copy numbers losses in 12p13.32 and 7p21.3 of 1.7 and 5.3 Mb in size, respectively. Among patients sharing microcephaly a common deleted region of ~ 300 kb in 12p13.32 locus containing two genes *CRACR2A* (HGNC: 28657) and *PRMT8* (HGNC: 5188) could be identified. Furthermore, based on a literature search on gene functions, expression patters, and phenotypic characteristics of published cases we have selected *PRMT8* as a promising candidate gene from common deleted region for further immunohistochemical (IHC) investigations on human post-mortem brain tissue.

## Methods

### Magnetic Resonance Imaging

Magnetic resonance imaging in the patient was performed on a 3T MR device (Magnetom TrioTim, Siemens; Germany), with 32-channel head coil, using standard set of sequences that included sagittal 3D magnetization prepared rapid acquisition gradient echo (MPRAGE) sequence with voxel size 1 × 1 × 1 mm (TR = 2,300 ms; TE = 3 ms; flip angle = 9 degrees; matrix: 256 × 256). Volumetric processing of MR images was conducted by using the CIVET (version 1.1.11) pipeline developed at Montreal Neurological Institute, Brain Imaging Center. The control group consisted of five female subjects with an average age of 22.7 ± 0.7 years who were scanned on the same MR device and volumetric analysis was performed using the same CIVET (version 1.1.11) pipeline.

### Cytogenetic and Molecular Cytogenetic Studies

Giemsa (GTG) based banding cytogenetics on chromosomes derived from patient peripheral blood lymphocytes, was done according to standard procedures. Genomic DNA was extracted from patients whole blood by Puregene DNA Purification Kit (Gentra Systems, Minneapolis, MN, USA) following manufacture instructions.

Multiplex ligation dependent probe amplification (MLPA) analysis with SALSA MLPA P245 Microdeletion Syndromes-1A probemix was performed according to the manufacturer's instructions (MRC-Holland, the Netherlands) using ABI-PRISM 3130XL Genetic Analyzer (Applied Biosystems, Foster City, USA). Data analysis was done by GeneMarker software package (SoftGenetics, USA).

Array comparative genomic hybridization (aCGH) analysis was performed on SurePrint G3 4X180K microarray (Agilent, US) according to manufacture instructions. Promega Human Genomic DNA was used as a control. Data analysis was carried out on CytoGenomics 4.0. software (Agilent, US) and GRCh38 assembly was used.

For affected genes: (1) brain specific expression were extracted from publicly available dataset with in house developed algorithm (GEO Accession viewer GSE25219)^1^; (2) GTEx Portal was applied to vizualize tissue-specific gene expression; (3) haploinsufficency (%HI) and pLI scores for affected genes were retrieve from Decipher. High ranks of %HI (e.g. 0–10%) indicate that a gene is more likely to exhibit haploinsufficiency, low ranks of %HI (e.g., 90–100%) suggest a gene to be less likely to exhibit haploinsufficiency. pLI score depicts the probability that a gene is intolerant to a heterozygous Loss of Function (LoF) mutation. The pLI score is the probability that a given gene falls into the haploinsufficient category, therefore is extremely intolerant for loss-of-function variation. Genes with high pLI scores (pLI ≥ 0.9) are extremely LoF intolerant, whereby genes with low pLI scores (pLI ≤ 0.1) are LoF tolerant; 4) Mouse Genome Informatics (MGI) database was screened for fitting mouse models; 5) PubMed, ClinVar, Decipher, GeneCards and Genomic Variants in Human Genome (Build GRCh37: Feb. 2009, hg19) (Gold Standard) databases were searched for comparable literature data.

### Immunohistochemically Analysis of *PRMT8* Gene

Immunohistochemisty was performed using histological sections from fixed, post-mortem fetal, preterm and young adult brains ranging from 12 PCW (postconceptional weeks) to 11 years. Examined brains are part of Zagreb Neuroembryological Collection and University of Maryland Brain and Tissue Bank. Brain specimens were obtained from medically indicated or spontaneous abortions at several clinical and pathological departments of the University of Zagreb, School of Medicine, Zagreb, Croatia. Informed consent was provided, and procedures were approved by the corresponding Institutional Review Boards. Fetal age was estimated on the basis of crown-rump length (CRL, in mm) and pregnancy records. All specimens that went through genetic analysis and did not show any abnormalities were classified as normal control material. Brains were fixed by immersion in 4% paraformaldehyde in 0.1 M phosphate-buffered saline (PBS, pH 7.4) and tissue blocks were either frozen or embedded in paraffin wax. For IHC staining, following deparaffinization and pre-treatments with 0.3% hydrogen peroxide and blocking solution, sections were incubated with the primary antibody anti-PRMT8 (LifeSpan Biosciences, (C-Terminus) LS-C328308, Rabbit polyclonal) which was applied in a blocking solution in a ratio of 1:50. Secondary biotinylated anti-rabbit antibody and tertiary complex from Vectastain ABC kit (Vector Laboratories, Burlingame, CA, USA) was used according to the manufacturer protocol. Visualization of the staining was done with Sigmafast DAB with metal tablets (Sigma-Aldrich Co. LLC) according to the manufacturer protocol. Stained sections were embedded with coverslipes and Polymount (Polysciences). Sections were scanned by the high-resolution digital slide scanner NanoZoomer 2.0RS, NDP.view2 software (Hamamatsu, Japan). According to literature data on *PRMT8* expression pattern coronal sections were scanned and examine in detail. Two samples for each time period were analyzed and each experiment was repeated twice.

## Results

### Clinical Report on Patient

**Figure 1:**
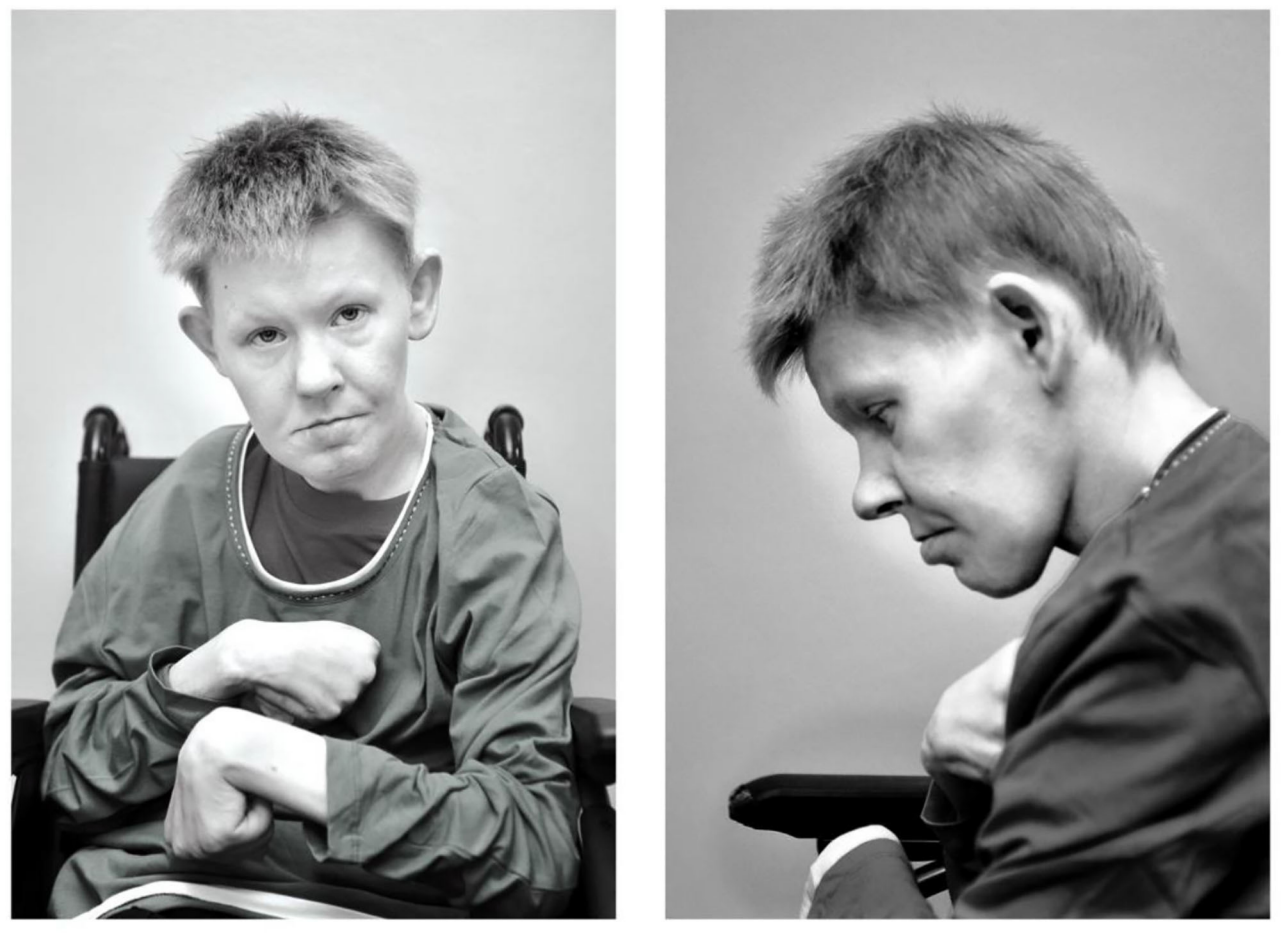
The patient at the age of 34 years.

At time of diagnosis, the patient was a 34 years old woman with unexplained intellectual disability (ID) (Figure 1). There were no reliable data on the course of her early development. At the age of 3 years, she has been placed in a foster care home, and at that time was diagnosed with ID, microcephaly, encephalopathy, and epilepsy. Physical examination also revealed strabismus, otapostasis, hypoacusis, scab covered skin below ears (because of the stereotype hand movements), autistic behavior, undeveloped speech and psychomotor restlessness. The patient was unaware of the environment, and therefore, verbal or social contacts were impossible to establish. According to medical documentation, stereotypic hand movements present as continuous movements toward the face could not be controlled by any medication. At the age of 34 years the patient was fully dependent on wheel chair and displayed an asthenic build up with a bent stature, scoliosis, contractures of shoulders and elbows with enhanced spasticity, chest deformity and joint laxity. Her height was 136 cm (>5th percentile), and weight 37 kg (>5th percentile). At the time of genetic evaluation, secondary sex characteristics were not developed and the patient could not control her sphincter.

### Magnetic Resonance Imaging on Patient

On magnetic resonance exam performed by standard set of triplane sequences head circumference was 49.5 cm, a value that fits to criteria of microcephaly. Standard MR examination shows normal organization of gyri and sulci, centrally located ventricular system of regular size, appropriate morphology of optic chiasm, cavernous sinuses, and pontocerebellar angles. No signs of acute ischemia and hemorrhage or focal expansive process were found. Cervical segment available for analysis showed spinal canal stenosis, most pronounced at the level of the C5 vertebra; the anteroposterior diameter was 8 mm. Volumetric analysis using the program CIVET (version 1.1.11) pipeline12 showed a marked reduction in total intracranial volume to a value of 750.18 cm^3^ (control group 1,380.41 ± 131.08 cm^3^), of which 290.17 cm^3^ was of gray matter (control group 700.91 ± 64.41 cm^3^), 406.00 cm3 was of white matter (control group 527.49 ± 65.40 cm^3^) and 56.01 cm^3^ was of cerebrospinal fluid (control group 152.00 ± 11.67 cm^3^). The performed volumetric analysis therefore shows a pronounced reduction in the size of the brain parenchyma, primarily due to the reduction of gray matter.

### Cytogenetic and Molecular Cytogenetic Studies on Patient

Banding cytogenetics and MLPA analysis were compatible with a normal female karyotype (data not shown).

By aCGH, copy number losses in 7p21.3 (chr7:7984615-13262873, GRCh38, 5.3 Mb) and in 12p13.32 (chr12: 3398438−5112197, GRCh38, 1.7 Mb) were identified as most likely clinically significant pathogenic aberrations. Detail summary for all affected genes from pathogenic regions is available in Supplements 1 and 2.

**Figure 2:**
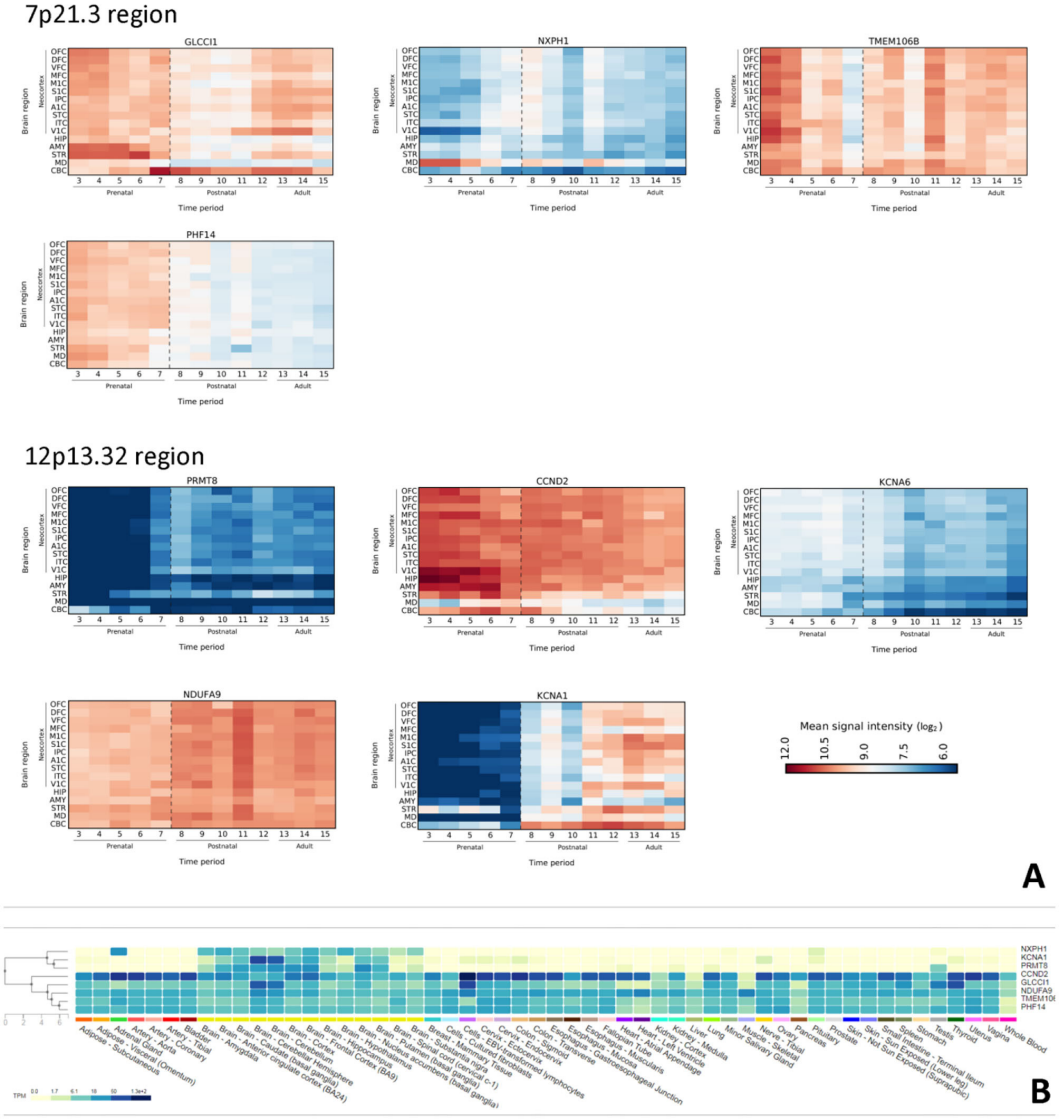
**(A)** Spatiotemporal expression profile of selected genes in the human brain from 7p21.3 and 12p13.32 regions. On Y-axis is presented expressions intensity by log_2_ ration, as in a scale bare. X-axis, numbers are representing time period (OFC, Orbital prefrontal cortex; DFC, Dorsolateral prefrontal cortex; VFC, Ventrolateral prefrontal cortex; MFC, Medial prefrontal cortex; M1C, Primary motor (M1) cortex; S1C, Primary somatosensory (S1) cortex; IPC, Posterior inferior parietal cortex; A1C, Primary auditory (A1) cortex; STC, Superior temporal cortex; ITC, Inferior temporal cortex; ITC, Inferior temporal cortex; V1C, Primary visual (V1) cortex; HIP, Hippocampus; AMY, Amygdala; STR, Striatum; MD, Mediodorsal nucleus of the thalamus; CBC, Cerebellar cortex. **(B)** GTEx database search for brain expressed genes. Note that first three lines are *NXPH1, KCNA1*, and *PRMT8* genes whose expression is largely restricted to the brain.

Based on spatio-temporal transcriptome of the human brain *GLCCI1, NXPH1, PHF14, TMEM106B* (from 7p21.3), and *PRMT8, CCND2, NDUFA9, KCNA1, KCNA6* (from 12p13.32) were detected as brain expressed genes. *GLCCI1* is highly expressed in prenatal brain, especially during early fetal and early midfoetal time in prefrontal cortex and basal ganglia. On a contrary, expression level goes up neonatally and remains stable in cerebellum throughout postnatal period and adulthood. *NXPH1* expression in neocortex is stable through lifetime and reaches the highest level neonatally. *PHF14* is highly expressed prenatally in all cortical and subcortical regions. Neonatally its expression gradually degrades, and is low from infancy onwards. *TMEM106B* microarray expression data shows highest expression level at early fetal and early mid-fetal period in neocortex, and in all subcortical structures at the time of intense proliferation and other neurodevelopmental processes. Its expression reaches the lowest expression level during late fetal period, but overall gets high again postnatally. *PRMT8* expression level is low prenatally, goes up around neonatal period and remains high throughout adulthood. *CCND2* showed highest expression levels from early to late fetal period in neocortex and in subcortical structures, especially during the time of important neurodevelopmental processes, such as proliferation; it remains high postnatally. Lowest expression levels are detected in thalamus throughout lifetime. *NDUFA9* shows relatively high expression level prenatally and postnatally in all examined brain regions, including all neocortical areas. Interestingly enough, it reaches an expression peak during childhood. *KCNA1* expression level is low prenatally, goes up around neonatal period and remains high throughout adulthood. *KCNA6* expression in all examined brain regions is stable prenatally and neonatally; gradually expression is getting lower postnatally through adulthood (Figure 2A).

Further investigation for aforementioned brain expressed genes in GTEx database reveal that *NXPH1, KCNA1*, and *PRMT8* are brain exclusively expressed genes (Figure 2B).

Highest ranks of %HI is reported for *NXPH1* (3,86) and *PHF14* (10,66) from 7p; while from 12p *CCND2* (2,99) and *PRMT8* (19,66) have highest rank; meaning that they are more likely to exhibit haploinsufficiency. Regarding pLI, *PRMT8* (0,98) and *CCND2* (0,99) are extremely LoF intolerant.

DGV Gold Standard Variants for affected regions reveal that 12p13.32 has less CNVs reported then 7p21.3 (Supplement 3).

Most commonly reported phenotypes for 7p21.3 deletions are: intellectual disability or global developmental delay, craniosynostosis, delayed speech and language development, brachydactyly, microtia, ptosis, seizures and single transverse palmar crease; while for 12p13.32 deletion there are: intellectual disability or global developmental delay, microcephaly, muscular hypotonia, scoliosis, small for gestational age, finger clinodactyly, short stature, strabismus and epicanthus (Table 1). List of all reported cases with phenotypes is available in Supplement 4.

**Table 1:** The prevalence of the most common clinical features reported from literature, database, and presented case.

	**Phenotype**	**Prevalence**
7p21.3	Intellectual disability	40%
	Craniosynostosis	14%
	Developmental delay and/or	12%
	other significant developmental or	
	morphological phenotypes (yes)	
	Delayed speech and language development	9%
	Brachydactyly	6%
	Microtia	6%
	Ptosis	6%
	Seizures	6%
	Single transverse palmar crease	6%
12p13.32	Intellectual disability	60%
	Microcephaly	26%
	Muscular hypotonia	13%
	Scoliosis	12%
	Small for gestational age	12%
	Finger clinodactyly	11%
	Short stature	11%
	Strabismus	11%
	Epicanthus	10%
	Micrognathia	10%

*In total 65 cases of 7p21.3 deletions were analyses and 82 cases of 12p13.32 deletions*.

MGI database search has indicate that genes from 12p13.32 could contribute to neurodevelopment in mouse, as mice homozygous for a knockout allele of *Prmt8, Ccnd2, Ndufa9, Kcna1*, and *Kcna6* all have diverse effects including behavioral abnormalities and nervous system phenotypes. List of all available genes from MGI database is in Supplement 5.

### Immunohistochemically Analysis of *PRMT8* Gene

**Figure 3:**
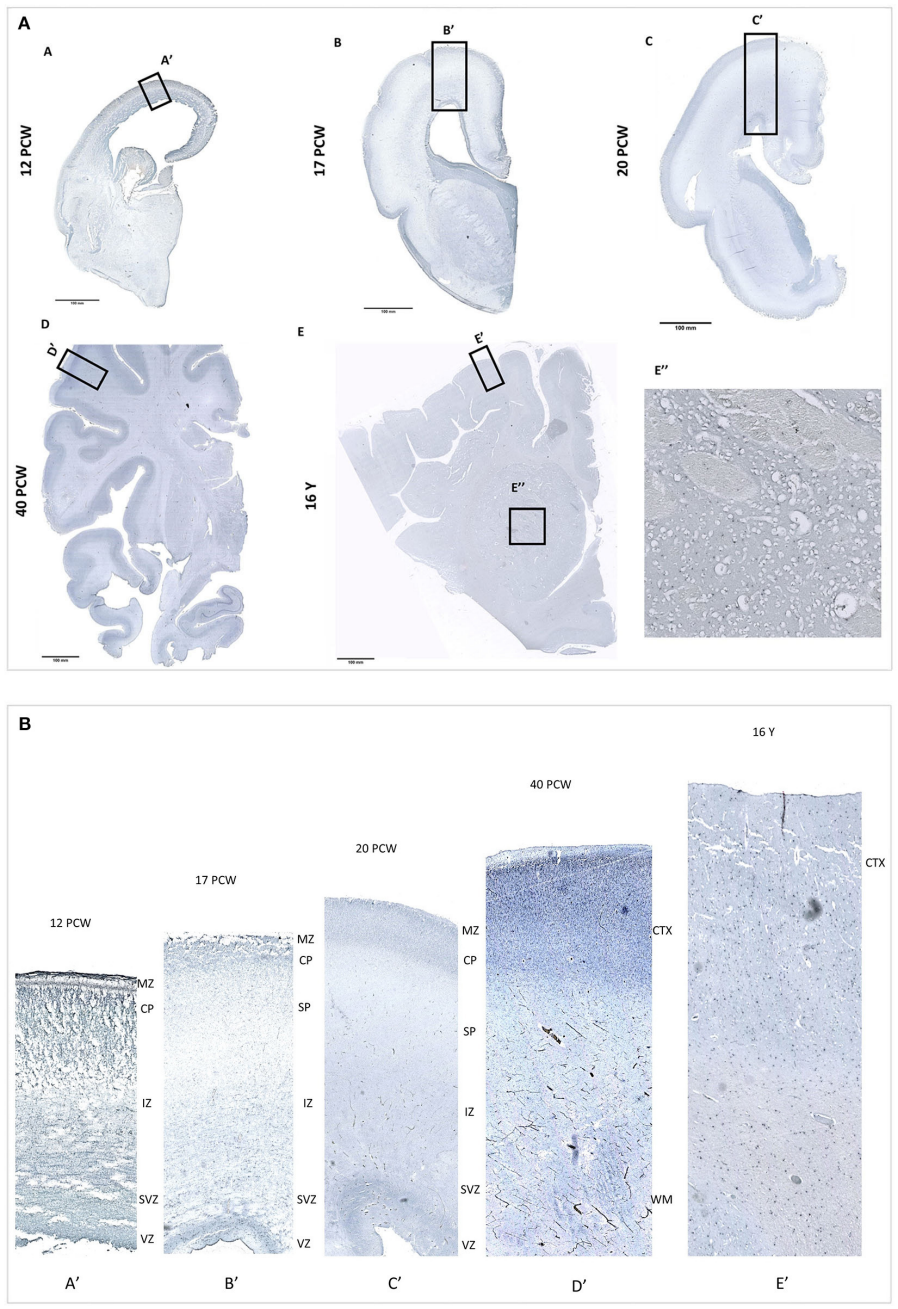
**(A)** PRMT8 immunoreactivity is shown on fetal [12 PCW (A), 17 PCW (B), 20 PCW (C)], newborn [(40 PCW (D)], and postnatal [16 years (E)] coronal sections of the human brain tissue. In addition, PRMT8 immunoreactive cells are found in the striatum throughout development, but especially intense in postnatal 16 y old specimen (E"). **(B)** Insets show enlarged corresponding cerebral cortex. During fetal development *PRMT8* immunoreactive cells are found in the cerebral cortex, especially in the proliferative ventricular zone (VZ) and the cortical plate (CP).

*PRMT8* expression patters showed that during fetal development *PRMT8* immunoreactive cells are found in the cerebral cortex, especially in the proliferative ventricular zone (VZ) and the cortical plate (CP). Furthermore, *PRMT8* immunoreactive cells are found in the striatum throughout development, but especially intense in postnatal 16 year old specimen (Figure 3). Obtained results are in corresponds to *PRMT8* expression pattern from database resource (Kang et al., 2011).

## Discussion

To the best of our knowledge, the presented case is unique in harboring two specific deletions in 7p21.3 and 12p13.32.

### 7p21.3 Region

To our knowledge only two postnatal and two prenatal cases of 7p21.3 deletions have been described in the literature (PubMed) (Shetty et al., 2007; Liao et al., 2012; Massalska et al., 2014). Additional 34 cases with described phenotype and deletion overlapping the affected region have been reported in Decipher. ClinVar database search for 7p21.3 cytogenetic band reorganizing 27 cases of deletions for which phenotypes are reported. Comparative evaluation of literature and databases for overlapping phenotypic features has showed that most commonly presented are intellectual disability/global developmental delay (40%), craniosynostosis (14%), and delayed speech and language development (9%). In 6% of case reported are observed brachydactyly, microtia, ptosis, seizures and single transverse palmar crease (Supplement 6).

Upon comparison of the expression data, gene functions, %HI and pLI scores, as well as described cases, out of all affected genes from 7p21.3 region most likely contributing to the observed phenotype are *NXPH1* and *TMEM106B* genes. Summary for all genes from 7p21.3 is available in Supplement 1.

Neurexophilin 1 (*NXPH1*, HGNC ID: 20693) is CNS specific genes, exclusively expressed in mediodorsal nucleus of the thalamus (https://www.gtexportal.org/home/). More specifically, *in situ* hybridization data suggested that prenatally it is expressed in migratory interneuron precursors and in select inhibitory interneurons of the adult brain (Petrenko et al., 1996; Batista-Brito et al., 2008). Additionally, mouse *Nxph1* serves as a ligand for synaptic cell adhesion molecules, α-Nrxn (α-neurexins) (Missler et al., 1998). On more subtitle level deletion of *Nxph1* in mouse impairs GABABR-mediated inhibition at GABAergic synapses in the nucleus reticularis thalami and at these synaptic terminals *Nxph1* is required for short-term plasticity (Born et al., 2014). These findings are important, as it is know that thalamocortical circuitry is responsible for functions like sleep–wake regulation, cognition and neuronal attention (Ichtchenko et al., 1995, 1996; Boucard et al., 2005; Reissner et al., 2014), all which are severely affected in present case and could be, at least in parts, explained by the haploinsufficency of *NXPH1* gene (%HI 3.86) In addition, we cannot rule out that deletion of *NXPH1* has effect on α-Nrxn, which are essential for transmission at synapses in the central and peripheral nervous system and by this having additional roles in synapse formation and differentiation (Reissner et al., 2014).

Transmembrane protein 106B (*TMEM106B*, HGNC ID: 22407) by its function is involved in dendrite morphogenesis and maintenance by regulating lysosomal trafficking via its interaction with *MAP6* (Microtubule Associated Protein 6). *TMEM106B* may inhibit retrograde transport of lysosomes along dendrites and it is required for dendrite branching (Brady et al., 2013; Schwenk et al., 2014). Mutation in *TMEM106B* are cause of leukodystrophy, hypomyelinating, 16 (MIM 617964) autosomal dominant neurologic disorder characterized by onset of hypotonia, nystagmus, mildly delayed motor development in infancy and degeneration of the white matter in the brain. Affected individuals have motor disabilities, including ataxic or broad-based gait, hyperreflexia, intention tremor, dysmetria, and a mild pyramidal syndrome. Some patients have cognitive impairment, whereas others may have normal cognition or mild intellectual disability with speech difficulties. Brain imaging typically shows hypomyelination, leukodystrophy, and thin corpus callosum. In the present case microcephaly is most likley due to reduction in the size of the brain parenchyma, primarily reduction of gray matter; thus, it is questionable to what extend monosomy of *TMEM106B* contributes to observed phenotype. Nevertheless, motor disabilities being present in our case could be explained, at least in parts, by monosomy of *TMEM106B*. Furthermore variants in *TMEM106B* may alter progranulin levels (Finch et al., 2011). Mutations in the progranulin precursor gene (*GRN*; HGNC ID: 4601) causing impaired production or secretion of progranulin are a common Mendelian cause of frontotemporal lobar degeneration with TDP-43 inclusions (FTLD-TDP; MIM 607485) (Van Deerlin et al., 2010). FTLD-TDP is a fatal neurodegenerative disease most commonly presents with social, behavioral, or language deterioration. We can only speculate about contribution of *TMEM106B* to observe phenotype as our proband was not old enough to develop symptoms of FTLD-TDP. In addition, recognizing signs of neurodegenerative disease could be challenging in presented case giving the severity of presented phenotype.

### 12p13.32 Region

**Figure 4:**
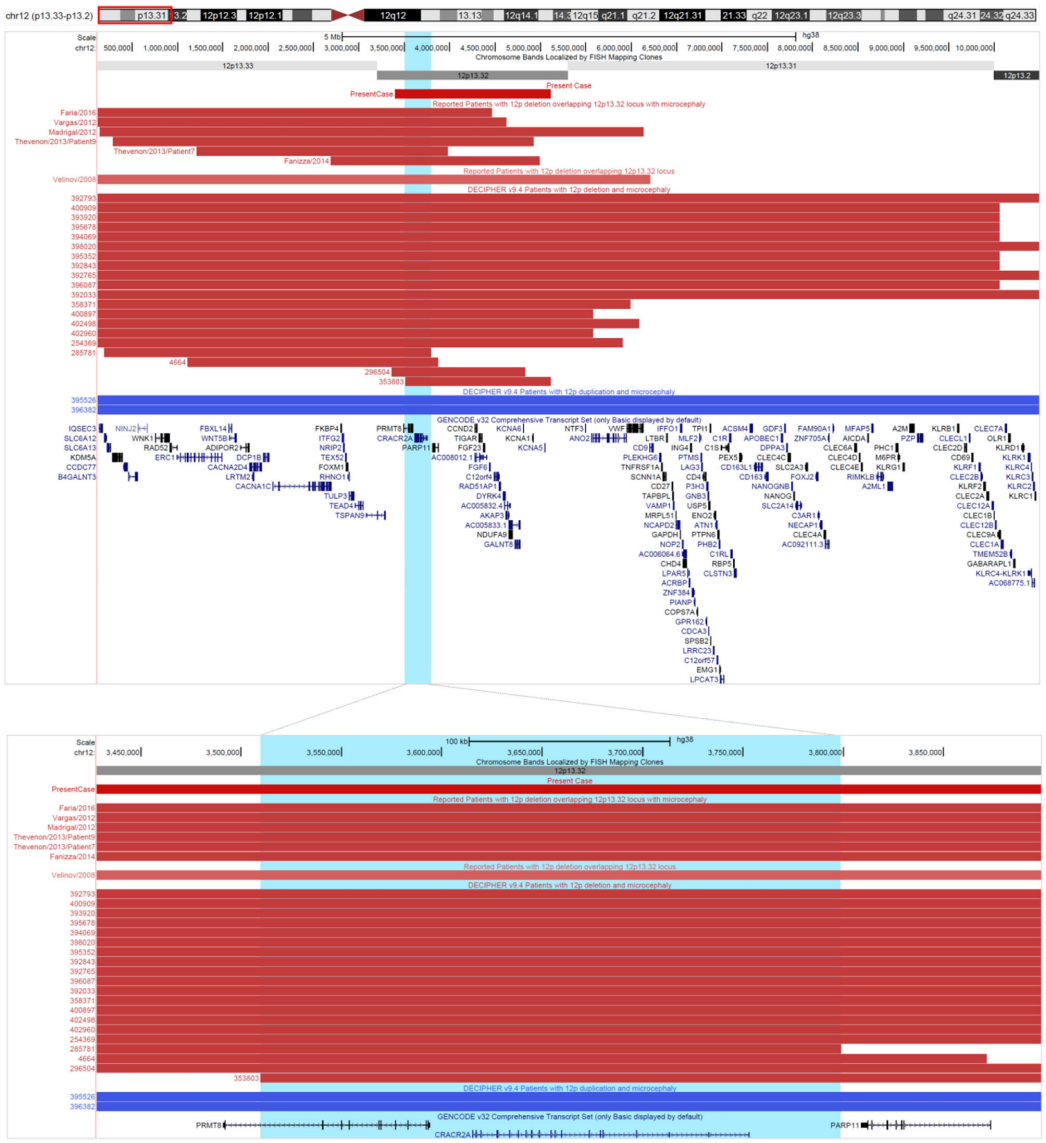
Comma deleted region in 12p. Cases of deletions (in red) and duplications (in blue) involving 12p13.32 locus, describer in literature and Decipher. Comma deleted region in 12p is highlighted in light blue. All coordinates are displayed in GRCh38/hg38 assembly. Reported are only cases with definitely and likely pathogenic deletions/duplications from Decipher.

Literature search for 12p13.32 deletions revealed seven previously published cases from PubMed (Velinov et al., 2008; Madrigal et al., 2012; Vargas et al., 2012; Thevenon et al., 2013; Fanizza et al., 2014; Faria et al., 2016). From Decipher there are 59 cases of deletions with reported phenotype, and additional 15 cases are found in ClinVar. Comparative phenotypic evaluation of literature and databases has showed that most commonly reported are intellectual disability or global developmental delay (60%), microcephaly (26%), muscular hypotonia (13%), scoliosis (12%), small for gestational age (12%). In 11% of the cases reported are finger clinodactyly, short stature and strabismus, while epicanthus and micrognathia are reported in 10% (Supplement 6). Among patients sharing microcephaly a common deleted region in 12p13.32 locus of ~ 300 kb containing two genes *CRACR2A* (HGNC: 28657) and *PRMT8* (HGNC: 5188) could be identified (Figure 4).

Upon comparison of the expression data, gene functions, %HI and pLI scores, as well as described cases, out of all affected genes from 12p13.32 region most likely contributors to observed phenotype are genes *PRMT8, CCND2*, and *KCNA1*. Summary for all genes from 12p13.32 is available in Supplement 2.

Cyclin D2 (*CCND2*, HGNC ID: 1583) belongs to a highly conserved cyclin family (*CCND1, CCND2*, and *CCND3*). They are all components of the core cell cycle machinery, but they exhibit distinct highly orchestrated tissue specific expression patterns which are controlled by extracellular environment (Matsushime et al., 1991; Ciemerych et al., 2002). *CCND2* has high expression levels in almost all brain regions and throughout lifetime. In developing brain *CCND1* and *CCND2* are the primary cyclins involved in G1/S transition, and their expression pattern is dynamically changing (Ross et al., 1996; Wianny et al., 1998; Glickstein et al., 2007a). Loss of either cD2 or cD1 in mouse results in smaller forebrains (microcephaly), but only *Ccnd2* null mice (*cD2*^−^*/*^−^) show selectively affected specific neuronal subtypes (Huard et al., 1999; Glickstein et al., 2007b). Recently disease-causing gain-of-function mutations in the *CCND2* gene was identified in patients with megalencephaly-polymicrogyria-polydactyly-hydrocephalus syndrome-3 (MPPH3; MIM 615938) (Mirzaa et al., 2014). According to the findings of Mirzaa et al. (2014) *CCND2* is a one of the major determinant of the brain size as it is necessary for geometric expansion of cellular output from the subventricular zone. Thus, if *CCND2* expression level is increased, cells will be maintained in cycle, which will lead to increasing numbery of progenitors and vice versa. Therefore, we hypothesize that haploinsufficiency of *CCND2* (%HI 2.32) is a strong candidate gene for reduced brain size in the presented case.

One gene from common deleted region is protein arginine methyltransferase 8 (*PRMT8*, HGNC ID: 5188). Arginine methylation is widespread post-translation modification involved in a number of cellular systems, including DNA repair, RNA transcription, signal transduction, protein compartmentalization and protein post-translational modification. Among nine PRMTs in mammal, *PRMT8* is unique because its expression is largely restricted to the brain (Figure 2B) and is the only membrane-bound PRMT via N-terminal myristoylation (Bedford and Richard, 2005; Lee et al., 2005). *PRMT8* also possesses the unusual property of acting both as methyltransferase and as phospholipase. Its phospholipase activity hydrolyses phosphatidylcholine to regulate dendritic morphology on one side and motor-coordinating behaviors and attention to another side (Kim et al., 2015; Simandi et al., 2015). *PRMT8* is also required for synaptic plasticity in the hippocampus and memory formation (Penney et al., 2017). Most recently it is demonstrated that *PRMT8* is present at neuronal synapses and its expression is upregulated in the hippocampus when dendritic spine maturation occurs (Lo et al., 2020). This was an important finding as spine maturation is essential for the stability of synapses and memory consolidation, and overproduction of the immature filopodia is associated with brain disorders. Mice homozygous for a *Prmt8* exhibit changes exclusively in behavior/neurological phenotype and nervous system structure (abnormal Purkinje cell dendrite morphology, hyperactivity, limb grasping and gait abnormalities, reduced levels of acetylcholine and choline along with increased phosphatidylcholine levels in the cerebellum) (Bult et al., 2019). In addition, our results on *PRMT8* immunoreactivity on human brain tissue reveal that during development *PRMT8* is especially expressed in the proliferative ventricular zone, zone of intense neurogenesis. Microcephaly in presented case is primarily consequence of reduction in gray matter volume, that can potentially be related to deprivation in *PRMT8* expression. Taken together, above findings are strongly suggestive that haploinsufficiency (%HI 19.66) of *PRTM8* contributes greatly to described neurological/motor impairments and microcephaly.

Potassium voltage-gated channels (Kv) is a big gene family with at least 40 genes divided into 12 subfamilies (KV1-Kv12) (Gutman et al., 2003). Among them, a large number has brain specific expression, primarily in neurons with a subset expressed in glial cells (Verkhratsky and Steinhäuser, 2000; Vacher et al., 2008). In presented case Potassium voltage-gated channel subfamily A member 1 and 6 genes (*KCNA1*, HGNC ID:6218; *KCNA6*, HGNC ID:6225) are deleted. Tissue specific expression profile for *KCNA1* shows that it is CNS specific genes, while data for *KCNA6* are not available (https://www.gtexportal.org/home/). Heterozygous mutations in *KCNA1* gene are responsible for episodic ataxia type 1 syndrome (EA1; OMIM # 160120) (Browne et al., 1994). Kv channels in neurons are important factors in setting of the resting membrane potential, modulating action potential frequency, and control of neurotransmitter release (Zhu et al., 2014). *Kcna1* homozygote knock-out mice exhibit diverse phenotypic changes including: behavioral abnormalities, megencephaly, and in one case, embryonic lethality; while *Kcna6* mice exhibit changes non-related to nervous system (an increased thermal nociceptive threshold and in females an increase in circulating triglyceride levels) (Bult et al., 2019). Despite high haploinsufficency score (*KCNA1* 30.34, *KCNA6* 44.08) it is likely that deletion of *KCNA1* and *KCNA6* contributes greatly to observed neurological impairments in presented case.

Contributions of other genes to the here observed phenotype cannot be ruled out, as there are no sufficient literature data. To elucidated contribution of aforementioned genes to microcephaly and neurological/motor impairments further studies and additionally cases are needed. In conclusion, we identified unique case harboring 5.3 Mb interstitial deletion in 7p21.3 and 1.7 Mb interstitial deletions in 12p13.32. The majority of literature data is suggestive that 12p13.32 deletion is responsible for major phenotypic features in presented case. Phenotypic variability observed in subjects with deletions overlapping 12p13.32 locus could be explained by size difference and gene content of affected region. Although observed phenotypic features cannot be definitely associated to a specific gene/genes from this two genomic locations, also two-hit- and compound heterozygosity-models cannot be ruled out, our conclusion is that haploinsufficency of genes from 12p13.32 region could provide strong candidate genes for microcephaly.

## Data Availability Statement

The original contributions presented in the study are included in the article/Supplementary Material, further inquiries can be directed to the corresponding authors.

## Ethics Statement

The studies involving human participants were reviewed and approved by Eticko povjerenstvo Medicinskoga fakulteta Sveučilišta u Zagrebu (04-1159-2006); Ravnateljstvo Centra za rehabilitaciju Stančić; Ministarstvo zdravstva i socijalne skrbi Vlade Republike Hrvatske (550-01/08-01/245). The patients/participants provided their written informed consent to participate in this study. Written informed consent was obtained from the individual(s) for the publication of any potentially identifiable images or data included in this article.

## Author Contributions

MRi did molecular cytogenetic analysis and drafted paper. MRa preformed MRI analysis. Immunohistochemisty was done by ZK and JK. Cytogenetic analysis was done by TL. All authors contributed to the article and approved the submitted version.

## Conflict of Interest

The authors declare that the research was conducted in the absence of any commercial or financial relationships that could be construed as a potential conflict of interest.

## Footnotes

^1^GEO Accession viewer GSE25219 Gene Expr. Omn. Available online at: https://www.ncbi.nlm.nih.gov/geo/query/acc.cgi?acc=GSE25219 (accessed December 9, 2020).

## Supplementary Material

Supplementary Table 1 Detail summary for affected genes from 7p21.3 pathogenic region.

Supplementary Table 2 Detail summary for affected genes from 12p13.32 pathogenic region.

Supplementary Table 3 Summary of MGI database search for genes from 7p21.3 and 12p13.32 region.

Supplementary Figure 1 DGV Gold Standard Variants for affected regions 12p13.32 and 7p21.3.

Supplementary Data Sheet 1 List of all reported cases with phenotypes from PubMed, Decipher, ClinVar for 7p21.3 and 12p13.32 region.

Supplementary Data Sheet 2 Most commonly presented phenotypic features for cases with 7p21.3 and 12p13.32 deletions.
