# Supplementary material for: 7p21.3 Together With a 12p13.32 Deletion in a Patient With Microcephaly—Does 12p13.32 Locus Possibly Comprises a Candidate Gene Region for Microcephaly?
Source: Front Mol Neurosci. 2021 Feb 4;14:613091. doi: 10.3389/fnmol.2021.613091 (PMC7890232; doi:10.3389/fnmol.2021.613091)
Supplement: Supplementary Table 1 — Detail summary for affected genes from 7p21.3 pathogenic region. [file Presentation_1.zip › Supplement 5. Summary of MGI database search for genes from 7p21.3 and 12p13.32 region.docx]

| **Band** | **Gene** | **Phenotype Summary** |
| --- | --- | --- |
| **7p21.3** | ***Nxph1*** | Mice homozygous for a targeted mutation show no obvious morbidity, premature mortality, or anatomical defects. However, males exhibit sterility and testis abnormalities probably because homologous recombination results in co-insertion of the 5' part of the HSV-TK cassette into the targeted locus. |
|  | ***Phf14*** | Mice homozygous for a knock-out allele exhibit complete neonatal lethality due to respiratory failure, pulmonary wall hypertrophy, abnormal sternum ossification, and increased proliferation of bone marrow-derived mesenchymal cells and mouse embryonic fibroblasts. |
|  | ***Tmem106B*** | Thrombocytosis, abnormal bone mineralization, abnormal circulating alkaline phosphatase level, abnormal epididymis morphology, decreased circulating insulin level, decreased mean platelet volume, enlarged epididymis, improved glucose tolerance increased circulating alkaline phosphatase level. |
| **12p13.32** | ***Prmt8*** | Mice homozygous for a knockout allele exhibit abnormal Purkinje cell dendrite morphology, hyperactivity, limb grasping and gait abnormalities, and show reduced levels of acetylcholine and choline along with increased phosphatidylcholine levels in the cerebellum. |
|  | ***Ccnd2*** | Homozygotes for a targeted null mutation are sterile: females lack a granulosa cell response to follicle stimulating hormone, while males have hypoplastic testes. Mutants also show decreased cerebellar granule cell and stellate neuron populations. |
|  | ***Ndufa9*** | Mice homozygous for a knock-out allele exhibit decreased embryo size, a rudimentary egg cylinder, failure of primitive streak formation, absent primitive node and head folds, failure to gastrulate, and complete lethality prior to organogenesis. |
|  | ***Kcna1*** | Various mutations at this allele have diverse affects including behavioral abnormalities, megencephaly, and in one case, embryonic lethality. |
|  | ***Kcna6*** | Homozygous mutation of this gene results in an increased thermal nociceptive threshold and in females an increase in circulating triglyceride levels. |
