# Supplementary material for: 7p21.3 Together With a 12p13.32 Deletion in a Patient With Microcephaly—Does 12p13.32 Locus Possibly Comprises a Candidate Gene Region for Microcephaly?
Source: Front Mol Neurosci. 2021 Feb 4;14:613091. doi: 10.3389/fnmol.2021.613091 (PMC7890232; doi:10.3389/fnmol.2021.613091)
Supplement: Supplementary Table 1 — Detail summary for affected genes from 7p21.3 pathogenic region. [file Presentation_1.zip › Supplement 1. Detail summary for affected genes from 7p21.3 pathogenic region.docx]

| Symbol | Gene name | HGNC | Molecular function | Brain expresed* | MalaCards diseases | Gene Ontology** | OMIM | Phenotype  MIM number | %HI | pLI |
| --- | --- | --- | --- | --- | --- | --- | --- | --- | --- | --- |
| GLCCI1 | glucocorticoid induced 1 | 18713 |  | yes |  |  | 614283 | 614400  Glucocorticoid therapy, response to | 40,81 | 0,06 |
| ICA1 | islet cell autoantigen 1 | 5343 | May play a role in neurotransmitter secretion | no |  | protein domain specific binding  membrane curvature sensor activity |  |  | 36,19 | 0,00 |
| NXPH1 | neurexophilin 1 | 20693 | May be signaling molecules that resemble neuropeptides  act by binding to alpha-neurexins and possibly other receptors | yes | paranoid schizophrenia  ADHD  constipation | signaling receptor binding | 604639 |  | 3,86 | 0,48 |
| NDUFA4 | NDUFA4 mitochondrial complex associated | 7687 | NDUFA4 is required for complex IV maintenance as one of the component of the cytochrome c oxidase | no | leigh syndrome with leukodystrophy  leigh syndrome  apolipoprotein c-iii deficiency | cytochrome-c oxidase activity | 603833 | 619065  Mitochondrial complex IV deficiency, nuclear type 21 | 45,68 | 0,02 |
| PHF14 | PHD finger protein 14 | 22203 |  | yes | floating-harbor syndrome  dandy-walker syndrome | Molecular function  protein and histon binding  contributes to histone acetyltransferase activity (H3-K23 specific)  metal ion binding |  |  | 10,66 | 0,27 |
| THSD7A | thrombospondin type 1 domain containing 7A | 22207 | Plays a role in actin cytoskeleton rearrangement. | no | Membranous Nephropathy  Osteoporosis  histiocytoid hemangioma |  | 612249 |  | 29,12 | 1,00 |
| TMEM106B | transmembrane protein 106B | 22407 | dendrite morphogenesis and maintenance  Required for dendrite branching  inhibiting retrograde transport of lysosomes along dendrites | yes | leukodystrophy, hypomyelinating, 16  pick disease of brain  semantic dementia  progressive non-fluent aphasia  frontotemporal dementia | Molecular function  Protein binding | 613413 | 617964  Leukodystrophy, hypomyelinating, 16 | 27,11 | 0,33 |
| VWDE | von Willebrand factor D and EGF domains | 21897 |  | no | hypertrophy of breast | calcium ion binding  signaling receptor binding |  |  | 77.80 | 0,00 |
| SCIN | scinderin | 21695 | regulatory function in exocytosis by affecting the organization of the microfilament network underneath the plasma membrane | no | Carbuncle  Ataxia, Sensory, 1, Autosomal Dominant | calcium ion binding and actin filament binding | 613416 |  | 52,68 | 0,00 |
| ARL4A | ADP ribosylation factor like GTPase 4A | 695 | cycles between an inactive GDP-bound and an active GTP-bound form | no |  | GTP binding and GTPase activity | 604786 |  | 44,54 | 0,81 |

* according to data from Kang et al.

** according to GeneCards

Haploinsufficency score (%HI) and pLI score were retrieve from Decipher.

High ranks of %HI (e.g. 0-10%) indicate a gene is more likely to exhibit haploinsufficiency, low ranks (e.g. 90-100%) indicate a gene is more likely to not exhibit haploinsufficiency.

pLI score indicates the probability that a gene is intolerant to a heterozygous Loss of Function (LoF) mutation. This analysis is based on high-quality exome data for 125,748 individuals of diverse ethnicities. The pLI score is the probability that a given gene falls into the Haploinsufficient category, therefore is extremely intolerant of loss-of-function variation. Genes with high pLI scores (pLI ≥ 0.9) are extremely LoF intolerant, whereby genes with low pLI scores (pLI ≤ 0.1) are LoF tolerant.
