# Supplementary material for: 7p21.3 Together With a 12p13.32 Deletion in a Patient With Microcephaly—Does 12p13.32 Locus Possibly Comprises a Candidate Gene Region for Microcephaly?
Source: Front Mol Neurosci. 2021 Feb 4;14:613091. doi: 10.3389/fnmol.2021.613091 (PMC7890232; doi:10.3389/fnmol.2021.613091)
Supplement: Supplementary Table 1 — Detail summary for affected genes from 7p21.3 pathogenic region. [file Presentation_1.zip › Supplement 2. Detail summary for affected genes from 12p13.32 pathogenic region.docx]

| Symbol | Gene name | HGNC | Molecular function | Brain expresed* | MalaCards diseases | Gene Ontology** | OMIM | Phenotype  MIM number | %HI | pLI |
| --- | --- | --- | --- | --- | --- | --- | --- | --- | --- | --- |
| PRMT8 | protein arginine methyltransferase 8 | 5188 | catalyze the formation of omega-N monomethylarginine (MMA) and asymmetrical dimethylarginine (aDMA) in proteins such as NIFK, myelin basic protein, histone H4, H2A and H2A/H2B dimer | yes | retinitis pigmentosa 22 | protein binding  methyltransferase activity  histone-arginine N-methyltransferase activity  S-adenosylmethionine-dependent methyltransferase activity  transferase activity | 610086 |  | 19,66 | 0,98 |
| THCAT155 | Thyroid Cancer-Associated Transcript 155 |  | RNA Gene | no |  |  |  |  |  |  |
| CRACR2A | calcium release activated channel regulator 2A | 28657 | plays a key role in store-operated Ca(2+) entry (SOCE) in T-cells by regulating CRAC channel activation | no | stormorken syndrome  myopathy, tubular aggregate | GTPase activity  calcium ion binding  protein binding  GTP binding  metal ion binding | 614178 |  |  |  |
| PARP11 | poly(ADP-ribose) polymerase family member 11 | 1186 | mediates mono-ADP-ribosylation of target proteins  Plays a role in nuclear envelope stability and nuclear remodeling during spermiogenesis (By similarity) | no | arthrogryposis, renal dysfunction, and cholestasis 1 |  | 616706 |  | 36,72 | 0,00 |
| CCND2 | cyclin D2 | 1583 | Regulatory component of the cyclin D2-CDK4 (DC) complex that phosphorylates and inhibits members of the retinoblastoma (RB) protein family including RB1 and regulates the cell-cycle during G(1)/S transition | yes | megalencephaly-polymicrogyria-polydactyly-hydrocephalus syndrome 3  megalencephaly-polymicrogyria-polydactyly-hydrocephalus syndrome 1  megalencephaly  hydrocephalus  polymicrogyria | protein kinase activity  protein binding  cyclin-dependent protein serine/threonine kinase regulator activity  protein kinase binding | 123833 | 615938  Megalencephaly-polymicrogyria-polydactyly-hydrocephalus syndrome 3 | 2,32 | 0,99 |
| TIGAR | TP53 induced glycolysis regulatory phosphatase | 1185 | Acts as a negative regulator of glycolysis  Contributes to the generation of reduced glutathione - correlating with its ability to protect cells from oxidative or metabolic stress-induced cell death  Plays a role in promoting protection against cell death during hypoxia  Plays a role in adult intestinal regeneration  Plays a neuroprotective role against ischemic brain damage  Plays a role in cancer cell survival by promoting DNA repair | no | Hypoxia  hemangioma of spleen | catalytic activity  bisphosphoglycerate 2-phosphatase activity  fructose-2,6-bisphosphate 2-phosphatase activity  protein binding  hydrolase activity | 610775 |  | 0,00 | 0,73 |
| FGF23 | fibroblast growth factor 23 | 3680 | Regulator of phosphate homeostasis. Inhibits renal tubular phosphate transport  Regulator of vitamin-D metabolism. Negatively regulates osteoblast differentiation and matrix mineralization | no | hypophosphatemic rickets, autosomal dominant  tumoral calcinosis, hyperphosphatemic, familial, 2  tumoral calcinosis, hyperphosphatemic, familial, 1  hyperphosphatemia  hypophosphatemia | fibroblast growth factor receptor binding  type 1 fibroblast growth factor receptor binding  protein binding  growth factor activity | 605380 | 193100  Hypophosphatemic rickets, autosomal dominant  617993  Tumoral calcinosis, hyperphosphatemic, familial, 2 | 63,57 | 0,03 |
| FGF6 | fibroblast growth factor 6 | 3684 | Plays an important role in the regulation of cell proliferation, cell differentiation, angiogenesis and myogenesis, and is required for normal muscle regeneration | no | apert syndrome  breast cancer  desmoid disease, hereditary | growth factor activity | 134921 |  | 24,44 | 0,03 |
| C12orf4 | chromosome 12 open reading frame 4 | 1184 | Plays a role in mast cell degranulation | yes | mental retardation, autosomal recessive 66  attention deficit-hyperactivity disorder  alacrima, achalasia, and mental retardation syndrome  autosomal recessive non-syndromic intellectual disability  distal arthrogryposis |  | 616082 | 618221  Mental retardation, autosomal recessive 66 | 44,66 | 0,00 |
| RAD51AP1 | RAD51 associated protein 1 | 16956 | May participate in a common DNA damage response pathway associated with the activation of homologous recombination and double-strand break repair | no | fanconi anemia, complementation group a | DNA binding  double-stranded DNA binding  single-stranded DNA binding  RNA binding  protein binding | 603070 |  | 57,70 | 0,00 |
| DYRK4 | dual specificity tyrosine phosphorylation regulated kinase 4 | 3095 | Possible non-essential role in spermiogenesis | no | mental retardation, autosomal dominant 7 | nucleotide binding  protein kinase activity  protein serine/threonine kinase activity  protein serine/threonine/tyrosine kinase activity  protein tyrosine kinase activity | 609181 |  | 57,06 | 0,00 |
| AKAP3 | A-kinase anchoring protein 3 | 373 | may be involved in oocyte recognition,sperm-oocyte binding protein | no | atrophy of prostate  male infertility  ovarian cancer  primary ciliary dyskinesia | protein kinase A binding  protein binding | 604689 |  | 72,87 | 0,06 |
| NDUFA9 | NADH:ubiquinone oxidoreductase subunit A9 | 7693 | Accessory subunit of the mitochondrial membrane respiratory chain NADH dehydrogenase (Complex I), that is believed not to be involved in catalysis. Required for proper complex I assembly | yes | mitochondrial complex i deficiency, nuclear type 26  leigh syndrome with leukodystrophy  liver disease  listeriosis | catalytic activity  NADH dehydrogenase activity  protein binding  NADH dehydrogenase (ubiquinone) activity  protein-containing complex binding | 603834 | 618247  Mitochondrial complex I deficiency, nuclear type 26 | 63,74 | 0,00 |
| GALNT8 | polypeptide N-acetylgalactosaminyltransferase 8 | 4130 | Probably catalyzes the initial reaction in O-linked oligosaccharide biosynthesis, the transfer of an N-acetyl-D-galactosamine residue to a serine or threonine residue on the protein receptor | no |  | polypeptide N-acetylgalactosaminyltransferase activity  ion channel activity  voltage-gated potassium channel activity  protein binding  transferase activity | 606250 |  | 88,43 | 0,00 |
| KCNA6 | potassium voltage-gated channel subfamily A member 6 | 6225 | Voltage-gated potassium channel that mediates transmembrane potassium transport in excitable membranes. | yes | episodic ataxia | ion channel activity  voltage-gated ion channel activity  voltage-gated potassium channel activity  delayed rectifier potassium channel activity  potassium channel activity | 176257 |  | 44,08 | 0,52 |
| KCNA1 | potassium voltage-gated channel subfamily A member 1 | 6218 | Voltage-gated potassium channel that mediates transmembrane potassium transport in excitable membranes, primarily in the brain and the central nervous system, but also in the kidney  Contributes to the regulation of the membrane potential and nerve signaling, and prevents neuronal hyperexcitability | yes | episodic ataxia, type 1  isolated autosomal dominant hypomagnesemia, glaudemans type  episodic kinesigenic dyskinesia 1  early infantile epileptic encephalopathy  episodic ataxia | ion channel activity  voltage-gated ion channel activity  voltage-gated potassium channel activity  delayed rectifier potassium channel activity  potassium channel activity | 176260 | 160120  Episodic ataxia/myokymia syndrome | 30,34 | 0,08 |
| KCNA5 | potassium voltage-gated channel subfamily A member 5 | 6224 | Voltage-gated potassium channel that mediates transmembrane potassium transport in excitable membranes. | no | atrial fibrillation, familial, 7  familial atrial fibrillation  atrial fibrillation  insulinoma  pulmonary hypertension | signaling receptor binding  ion channel activity  voltage-gated ion channel activity  voltage-gated potassium channel activity  delayed rectifier potassium channel activity | 176267 | 612240  Atrial fibrillation, familial, 7 | 49,12 | 0,00 |

* according to data from Kang et al.

** according to GeneCards

Haploinsufficency score (%HI) and pLI score were retrieve from Decipher.

High ranks of %HI (e.g. 0-10%) indicate a gene is more likely to exhibit haploinsufficiency, low ranks (e.g. 90-100%) indicate a gene is more likely to not exhibit haploinsufficiency.

pLI score indicates the probability that a gene is intolerant to a heterozygous Loss of Function (LoF) mutation. This analysis is based on high-quality exome data for 125,748 individuals of diverse ethnicities. The pLI score is the probability that a given gene falls into the Haploinsufficient category, therefore is extremely intolerant of loss-of-function variation. Genes with high pLI scores (pLI ≥ 0.9) are extremely LoF intolerant, whereby genes with low pLI scores (pLI ≤ 0.1) are LoF tolerant.
