# Supplementary figures and images for: 7p21.3 Together With a 12p13.32 Deletion in a Patient With Microcephaly—Does 12p13.32 Locus Possibly Comprises a Candidate Gene Region for Microcephaly?
Source: Front Mol Neurosci. 2021 Feb 4;14:613091. doi: 10.3389/fnmol.2021.613091 (PMC7890232; doi:10.3389/fnmol.2021.613091)

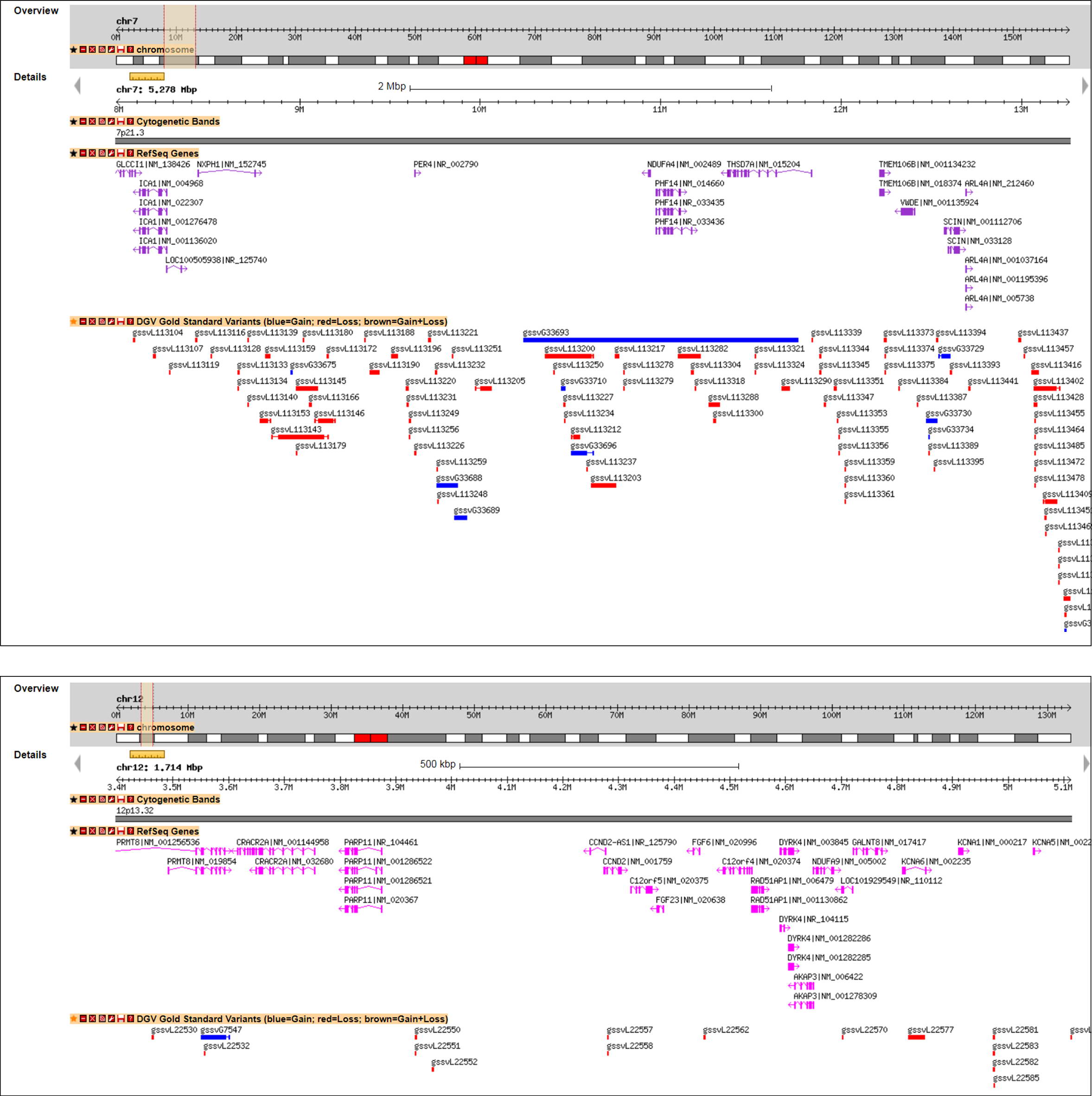

Supplement: Supplementary Table 1 — Detail summary for affected genes from 7p21.3 pathogenic region. [file Presentation_1.zip › Supplement 3. DGV Gold Standard Variants for affected regions 12p13.32 and 7p21.3.jpg]
